# Renal T1 Times on Cardiac Magnetic Resonance Reflect Renal Dysfunction and Are Associated with Adverse Outcomes: Insights from an All-Comer Cohort

**DOI:** 10.3390/jcm14010154

**Published:** 2024-12-30

**Authors:** Laura Lunzer, Carolina Donà, Katharina Mascherbauer, Christina Kronberger, Christian Nitsche, Matthias Koschutnik, Michael Poledniczek, Paul Felix Harbich, Christoph Kaufmann, Edita Pogran, Heda Kvakan, Dietrich Beitzke, Christian Loewe, Alexander Geppert, Christian Hengstenberg, Andreas Anselm Kammerlander

**Affiliations:** 1 Medical Department, Division of Cardiology, Medical University of Vienna, 1090 Vienna, Austria; laura.lunzer@gesundheitsverbund.at (L.L.); katharina.mascherbauer@meduniwien.ac.at (K.M.); christina.kronberger@meduniwien.ac.at (C.K.); christian.nitsche@meduniwien.ac.at (C.N.); matthias.koschutnik@meduniwien.ac.at (M.K.); michael.poledniczek@meduniwien.ac.at (M.P.); christian.hengstenberg@meduniwien.ac.at (C.H.); andreas.kammerlander@meduniwien.ac.at (A.A.K.); 2 Medical Department, Division of Cardiology and Intensive Care Medicine, Klinik Ottakring, 1160 Vienna, Austria; paul-felix.harbich@extern.gesundheitsverbund.at (P.F.H.); christoph.kaufmann@gesundheitsverbund.at (C.K.); edita.pogran@gesundheitsverbund.at (E.P.); heda.kvakan@gesundheitsverbund.at (H.K.); alexander.geppert@gesundheitsverbund.at (A.G.); 3 Division of Cardiovascular and Interventional Radiology, Department of Biomedical Imaging and Image-Guided Therapy, Medical University of Vienna, 1090 Vienna, Austria; dietrich.beitzke@meduniwien.ac.at (D.B.); christian.loewe@meduniwien.ac.at (C.L.)

**Keywords:** cardiac magnetic resonance, cardiorenal syndrome, renal T1 times, chronic kidney disease, cardiovascular risk stratification

## Abstract

**Background:** Renal disease is common in patients with cardiovascular disease (CVD) and is associated with adverse outcomes. Cardiac magnetic resonance (CMR) with advanced mapping techniques is the gold standard for characterizing myocardial tissue, and renal tissue is often visualized on these maps. However, it remains unclear whether renal T1 times accurately reflect renal dysfunction or predict adverse outcomes. **Aim:** To analyze the relationship between renal T1 times, renal dysfunction, and adverse outcomes. Adverse outcomes were defined as all-cause and cardiovascular death. **Methods:** Renal T1 times were measured in the native short-axis view in an all-comers cohort undergoing CMR. Renal function parameters were assessed at the time of CMR. **Results:** A total of 506 patients (mean age 60 ± 15 years, 53% male) were included in the analysis. A significant correlation was observed between log10 renal cortical T1 times and eGFR (r = −0.701, *p* < 0.001) and creatinine (r = 0.615, *p* < 0.001). Kaplan–Meier analysis showed an increased risk of all-cause (*p* < 0.001 by log-rank test) and cardiovascular mortality (*p* = 0.004 by log-rank test) in patients with renal cortical T1 times above the median. In the univariable Cox regression analysis, there was a significant association between renal cortical T1 times and increased risk of all-cause (HR = 1.73 [95% CI, 1.42–2.11] per every 100 ms increase *p* < 0.001) and cardiovascular mortality (HR = 1.41 [95% CI, 1.05–1.90] per every 100 ms increase, *p* = 0.021). This association remained statistically significant after adjustment for prespecified clinical factors (adjusted HR for all-cause death = 1.49 [95% CI, 1.10–2.02] per every 100 ms increase, *p* = 0.01; adjusted HR for cardiovascular death = 1.42 [95% CI, 1.05–1.90] per every 100 ms increase, *p* = 0.021). **Conclusions:** Our results indicate that there is a significant association between increased renal cortical T1 times and impaired renal function, as well as an increased risk of all-cause and cardiovascular mortality, although it should be noted that our results are preliminary and need to be validated in external cohorts performing renal biopsies.

## 1. Introduction

The impact of chronic kidney disease (CKD) on global morbidity and mortality is rapidly increasing, and CKD is a common comorbidity in patients with cardiovascular disease (CVD) [1]. The reciprocal influence of cardiovascular and renal diseases is reflected in the definition of cardiorenal syndrome (CRS) [2,3]. Three pathophysiological mechanisms are currently considered to be responsible for the development of cardiorenal or renocardial syndrome. These include (1) hemodynamic changes associated with low output syndrome or venous return; (2) dysregulation of the neurohumoral axis via sympathetic activation and activation of the renin–angiotensin–aldosterone system; and (3) other factors including systemic inflammation, changes in cell-mediated immunity, and metabolic changes such as malnutrition, anemia, and changes in bone metabolism [4]. In addition, the overlap between cardiorenal syndrome and cardiometabolic syndrome has led to the definition of cardiovascular–kidney–metabolic syndrome [5,6,7].

In clinical practice, serum creatinine and estimated glomerular filtration rate (eGFR) are the most commonly employed parameters for the assessment of renal dysfunction [4,8]. Nevertheless, a renal biopsy is indispensable for identifying the underlying pathology and differentiating between acute and chronic changes, including the extent of fibrotic changes present, which is also of prognostic value. Given the invasive nature of the procedure and the associated, albeit minor, bleeding complications, there are increasing efforts to identify non-invasive alternatives [9].

CMR with its advanced mapping techniques, including T1 mapping, is the accepted gold standard for non-invasive assessment of tissue composition. While T1 mapping, which allows for non-invasive tissue characterization, is well established in cardiac disease, little is currently known about its value in renal disease [10]. The two main pathologies reflected by increased T1 times are edema (an increase in tissue water, e.g., associated with acute infarction or inflammation) and an increase in interstitial space (e.g., fibrosis, scarring, or amyloid deposition) [11,12]. As renal T1 mapping may provide a more comprehensive and non-invasive approach for risk stratification in patients with cardiovascular and chronic kidney diseases, beyond the scope of laboratory markers, there is a need to integrate non-invasive tissue characterization techniques such as mapping sequences into routine clinical practice, thus facilitating a more comprehensive diagnostic assessment in complex patient populations.

A few studies have already reported on renal T1 mapping and its connection with renal function parameters with some preliminary promising results, but to date there are no outcome studies in patients with elevated renal T1 times. Furthermore, most of these studies were conducted on relatively small and specific patient groups [13,14,15,16,17].

Accordingly, the aim of this study was to examine the correlation between renal T1 times, renal dysfunction, and adverse outcomes in an all-comer cohort for CMR [18].

## 2. Methods

### 2.1. Study Design

This retrospective analysis was conducted within the scope of a prospective registry of patients at the Medical University of Vienna, Department of Internal Medicine II, Division of Cardiology. The study was approved by the Ethics Committee of the Medical University of Vienna (EK#2036/2015) and was implemented in compliance with the Declaration of Helsinki. Prior to their inclusion, all patients provided written informed consent.

The present study was conducted on an all-comer cohort undergoing CMR. Patients who were referred for CMR between 2013 and 2023 and for whom the kidneys were visible on short-axis T1 mapping sequences were included in the study. The referral diagnoses included valvular heart disease (49.8%), storage disease (13.4%), coronary artery disease (6.3%), heart failure (11.3%), myocarditis (3.6%), and other causes (15.6%). Patients with known cardiac amyloidosis were excluded from the study due to the potential for renal involvement, as the presence of amyloids may also increase T1 times and thus confound the data. Outpatients were also excluded from the study because their renal function parameters were not known. In addition, images with poor quality or with artefacts were excluded from our study.

Blood samples were collected from all subjects prior to CMR, including renal function parameters (serum creatinine and eGFR). The eGFR was calculated using the Cockcroft–Gault formula [19]. CKD stages were assigned using the KDIGO classification, although it should be noted that albuminuria categories could not be defined due to the lack of missing albuminuria values in our database. Participants did not need to follow any special preparation procedures, such as fasting.

### 2.2. Cardiac Magnetic Resonance Imaging

All included patients underwent CMR examinations using a 1.5 T system scanner (Avanto FIT; Siemens Medical Solutions, Erlangen, Germany) according to standard protocols [20,21]. Steady-state free-precision images were used for cine imaging (repetition time (ms)/echo time (ms), 3.2/1.2; flip angle, 64°; voxel size, 1.4 × 1.4 × 6 mm^3^; matrix, 180 × 256 pixels). For late gadolinium enhancement imaging, segmented inversion recovery sequences (700/1.22; flip angle, 50°; voxel size, 1.4 × 1.4 × 8 mm^3^; matrix, 146 × 256 pixels) were performed at least 10 min after injection of 0.1 mmol/mL gadobutrol (Gadovist; Bayer Vital GmbH, Leverkusen, Germany). T1 mapping was performed using electrocardiographically triggered MOLLI with a 5(3)3 prototype (5 acquisition heartbeats followed by 3 recovery heartbeats and another 3 acquisition heartbeats) in short-axis and 4-chamber views. The parameters of the T1 sequence were as follows: start inversion time (TI), 120 ms; TI increment, 80 ms; reconstructed matrix size, 256 × 218; measured matrix size, 256 × 144 (phase encoding resolution, 66%; phase encoding field of view, 85%). T1 maps were acquired 15 min before and after contrast administration. In addition to the standard T1 maps, regions of interest including parts of the cortical and medullary kidney were defined.

### 2.3. Definition of Renal T1 Times

**Figure 1:**
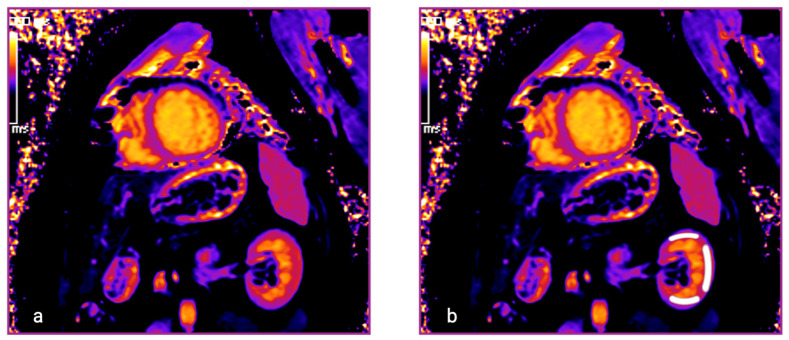
Example of a CMR short-axis view displaying T1 mapping in the left kidney (**a**,**b**). Three regions of interest were defined in the renal cortex, and renal T1 times are reported in milliseconds (ms) (**b**).

The renal native T1 times were determined in a T1 mapping short-axis view utilizing a dedicated workstation (IMPAX EE R20 XV, Agfa Healthcare) (Figure 1). On short-axis maps, three regions of interest (ROIs) were delineated manually in the upper, middle, and lower portions of the renal cortex. The corresponding T1 values were obtained for statistical analysis and expressed in milliseconds (ms). Intra- and interobserver variability was assessed in 20 randomly selected patients.

### 2.4. Outcome Measures

The primary aim of this study was to analyze the relationship between renal T1 times and renal functional parameters to provide evidence for a wider application of mapping sequences. The secondary objective was to evaluate the prognostic ability of renal T1 mapping. In the absence of normal values for renal T1 times, patients were stratified into two groups using median values for Kaplan–Meier analysis. We sought to use the median as an appropriate choice for analyzing results in situations where normal values are lacking, as it provides a robust, interpretable, and reliable measure of the central tendency of the data. Adverse outcomes were defined as all-cause or cardiovascular death.

To compile the mortality data, the Austrian national statistics authority (Statistics Austria) was queried. Furthermore, electronic health records were retrieved if available. Additionally, electronic health records were screened for clinical events.

### 2.5. Statistical Analysis

Continuous variables are expressed as means ± standard deviation or median with interquartile range. Categorical variables are expressed as percentages or totals. Comparisons of differences between variables between the two groups were made using the Mann–Whitney U-test for continuous variables and the Chi square test for categorical variables. In addition, log transformations were performed for renal cortical T1 times which were not normally distributed.

Spearman’s rank test was used to show the correlation between T1 times and renal function parameters. Kaplan–Meier curves with a corresponding log-rank test and univariable and multivariable Cox regression models were used for primary outcome analysis.

Firstly, all parameters were tested in a univariate model. Subsequently, parameters with a significant predictive value in the univariate Cox regression were included in a multivariate regression model with the median renal T1 times. For multivariable analysis, a prespecified set of risk factors was selected based on significant differences between the groups studied, including age, sex, NT-proBNP, hemoglobin, atrial fibrillation, and arterial hypertension.

Statistical analyses were performed with SPSS version 29.0, and a two-sided *p*-value ≤ 0.05 was considered significant. The Fine and Gray subdistribution hazard (SHR) model for estimating the cumulative incidence function (CIF) was implemented with Stata 15.

## 3. Results

The present study involved an investigation of 506 patients. The mean age of the patients was 60 ± 15 years, and 53% of those screened were male. The median eGFR was 66 mL/min/1.73 m² (IQR 17-178). As there are no existing normal values for renal T1 times, patients were stratified into two groups according to median values for comparison. The median renal cortical T1 times were 1090 ms (IQR 880–1361).

Table 1 shows the baseline characteristics and imaging parameters stratified by median renal cortical T1 times. The characteristics reflect typical features of patients with cardiorenal or cardiovascular–kidney–metabolic syndrome, with common comorbidities such as diabetes mellitus (19%), coronary artery disease (10%), atrial fibrillation (13%), arterial hypertension (47%), and hyperlipidemia (18%) (Table 1).

### 3.1. Correlation and Regression Analysis

**Figure 2:**
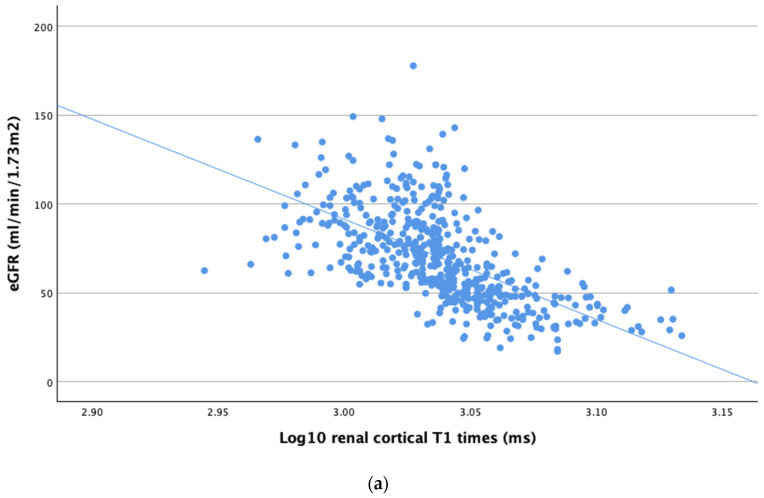

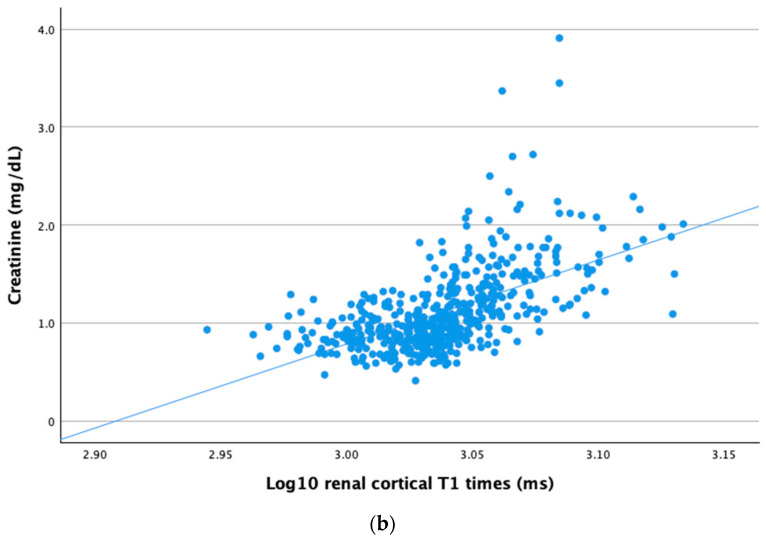
(**a**) Correlation between log10 renal cortical T1 times and eGFR. (**b**) Correlation between log10 renal cortical T1 times and creatinine.

The results showed a significant strong correlation between log10 renal cortical T1 times and eGFR (r = −0.701, *p* < 0.001, Figure 2a) as well as between log10 renal cortical T1 times and creatinine (r = 0.615, *p* < 0.001, Figure 2b). A significant correlation was also observed between log10 renal cortical T1 times and LVEF (r = −0.099, *p* = 0.035), RVEF (r = −0.104, *p* = 0.026), myocardial T1 times (r = 0.220, *p* < 0.001), age (r = 0.312, *p* < 0.001), extracellular volume (ECV) (r = 0.171, *p* = 0.016), hemoglobin (r = 0.314, *p* < 0.001), troponin T (r = 0.240, *p* < 0.001), and N-terminal pro-brain natriuretic peptide (NT-proBNP) (r = 0.342, *p* < 0.001).

Univariable regression analysis showed a significant association between log10 renal cortical T1 times and several clinical parameters, including LVEF (β = −0.101, *p* = 0.031), RVEF (β = −0.094, *p* = 0.05), eGFR (β = −0.612, *p* < 0.001), creatinine (β = 0.581, *p* < 0.001), myocardial T1 times (β = 0.176, *p* < 0.001), ECV (β = 0.158, *p* = 0.026), hemoglobin (β = −0.332, *p* < 0.001), and NT-proBNP (β = 0.249, *p* < 0.001) (see left side of Table 2). After adjustment for age and sex, the association remained significant for eGFR (β = −0.480, *p* < 0.001), creatinine (β = 0.532, *p* < 0.001), and hemoglobin (β = −0.194, *p* = 0.009) (see right side of Table 2).

### 3.2. Association Between Renal T1 Times and Outcome

A total of 193 patients died during follow-up [median, 48 months (IQR 24–72 months)]. Of these, 59 (31%) died from coronary heart disease, 19 from cancer (10%), 39 (20%) from heart failure events, 2 from amyloidosis (1%), 9 from infectious diseases (5%), 41 (23%) from other causes, and 23 (12%) from unknown causes.

**Figure 3:**
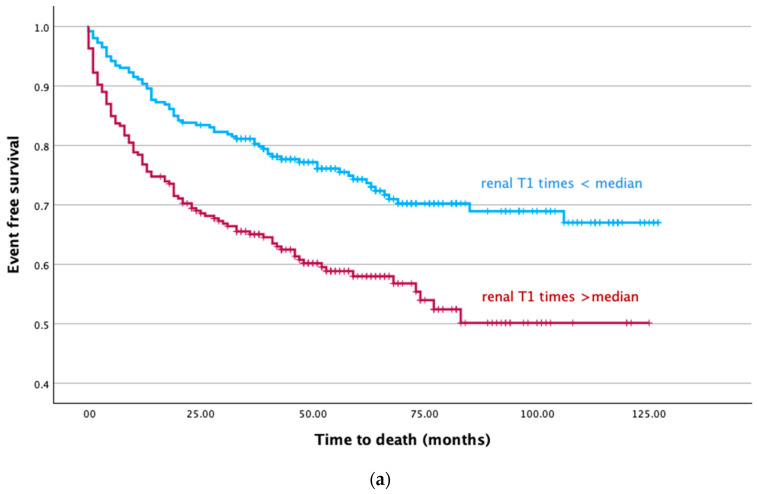

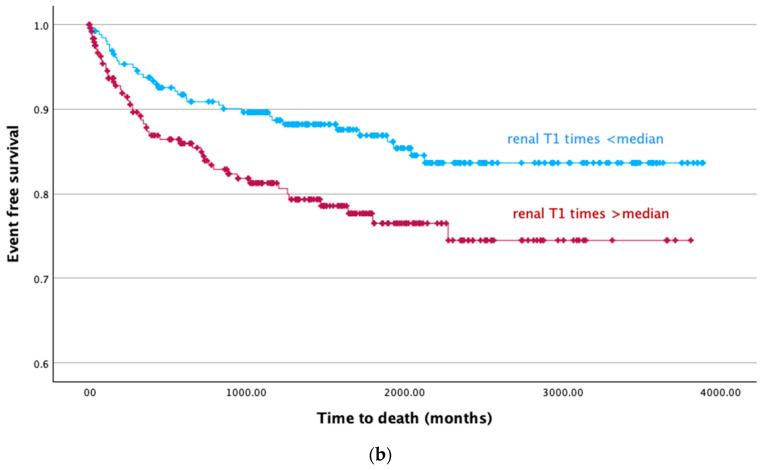
(**a**) Kaplan–Meier curves over time for all-cause death stratified by median T1 times. Group 1 (blue line, n = 260) includes patients with renal cortical T1 times below the median and Group 2 (red line, n = 246) includes patients with renal cortical T1 times above the median. There were 71 events in the group of patients with renal cortical T1 times below the median and 102 events in the group with renal cortical T1 times above the median. (**b**) Kaplan–Meier curves over time for cardiovascular death stratified by median T1 times. Group 1 (blue line, n = 260) includes patients with renal cortical T1 times below the median and Group 2 (red line, n = 246) includes patients with renal cortical T1 times above the median. There were 37 events in the group with renal cortical T1 times below the median and 53 events in the group with renal cortical T1 times above the median.

Kaplan–Meier analysis showed an increased risk of all-cause death in patients with renal cortical T1 times above the median (*p* < 0.001 by log-rank test) (Figure 3a,b). There were 71 events in the group of patients with renal cortical T1 times below the median and 102 events in the group with renal cortical T1 times above the median. Furthermore, Kaplan–Meier analysis showed an increased risk of cardiovascular death in patients with renal cortical T1 times above the median (*p* = 0.004 by log-rank test). There were 37 events in the group with renal cortical T1 times below the median and 53 events in the group with renal cortical T1 times above the median.

In the univariable Cox regression, renal cortical T1 times above the median were significantly associated with an increased risk of all-cause death (HR = 1.73 [95% CI, 1.42–2.11] per every 100 ms increase, *p* < 0.001) (Table 3). This association remained significant after adjustment for predefined clinical factors including age, sex, NT-proBNP, hemoglobin, atrial fibrillation, and arterial hypertension (adjusted HR = 1.49 [95% CI, 1.10–2.02] per every 100 ms increase, *p* = 0.01) (Table 3). With respect to the risk of cardiovascular mortality, the univariable regression model showed that renal cortical T1 times above the median were associated with an elevated risk (HR = 1.41 [95% CI, 1.05–1.90] per every 100 ms increase, *p* = 0.021) (Table 4). This association remained significant after adjustment for sex and arterial hypertension (adjusted HR = 1.42 [95% CI, 1.05–1.90] per every 100 ms increase, *p* = 0.021) (Table 4). Cox regression was not significant after further adjustment for NT-proBNP, age, atrial fibrillation, and hemoglobin (HR = 1.17 [95% CI, 0.76–1.78], *p* = 0.48).

We used the Fine and Gray subdistribution hazard (SHR) model for estimating the cumulative incidence function (CIF). When using non-CV death as a competing risk, the results were similar to our previously demonstrated analyses for renal T1 times per 100 ms increase (adjusted SHR = 1.62 [95% CI, 1.25–2.10], *p* < 0.001).

## 4. Discussion

The present study aimed to examine the correlation between renal functional parameters and renal cortical T1 times. The study yielded three principal findings. Firstly, the results of our study demonstrated that renal cortical T1 times are significantly correlated with renal function and can be employed to characterize renal tissue. Secondly, the results demonstrated a correlation between renal cortical T1 times and myocardial T1 times, indicating the presence of systemic fibrosis and the complex interaction between the cardiovascular and renal systems. Thirdly, our study is the first to demonstrate an association between elevated T1 times and all-cause and cardiovascular mortality, thus providing a new parameter for risk stratification in patients with cardiovascular and renal disease. Given the two endpoints assessed in our study, a competing risk analysis was performed, showing that the results using the Fine and Gray subdistribution hazard model (SHR) were similar to our previously demonstrated analyses for renal T1 times per 100 ms increase.

### 4.1. Extracardiac Applications of T1 Mapping

To date, only a limited number of studies have reported on renal T1 maps, with some preliminary findings that appear promising. The results of these studies indicate a significant correlation between elevated renal T1 times and compromised renal function (14–16).

Patients with CKD and healthy controls who underwent multiparametric magnetic resonance tomography, which combines T1 mapping and diffusion imaging, exhibited significantly different T1 times. There was a notable correlation between cortical and medullary T1 times and an association with eGFR [15]. These findings were corroborated by another study investigating the relationship between elevated renal T1 times and fibrosis in patients with IgA nephropathy, which showed significantly longer T1 times in patients with IgA nephropathy [16]. Furthermore, data indicate that renal T1 times are significantly prolonged following kidney transplantation, functioning as a marker of postoperative renal injury [14]. Another study demonstrated a strong correlation between renal T1 times and renal functional parameters, as well as a histologically confirmed correlation between elevated T1 times and the extent of fibrosis in a cohort of patients with chronic glomerulonephritis [13]. In addition, a recent study of 43,881 participants demonstrated increased T1 times in several organs, including the kidneys, as a marker of intestinal fibrosis [17]. Despite the differences in study protocols, the T1 times of patients with impaired renal function were consistently longer in all the studies, as well as in our own.

It is common for progressive renal disease to result in renal fibrosis, which may in turn lead to increased renal T1 times. It should be noted that inflammatory diseases such as interstitial nephritis may also lead to prolonged renal T1 times [22]. Given the established link between renal fibrosis, typically identified through renal biopsy, and unfavorable outcomes, renal T1 mapping may provide a more comprehensive and non-invasive approach for risk stratification in patients with cardiovascular and chronic kidney disease. Therefore, it is important to validate our preliminary study results in external cohorts performing renal biopsies to correlate histological results with renal T1 times and to exclude other causes that may lead to increased renal T1 times, such as inflammatory diseases.

It should also be noted that our analysis was based on renal T1 times above the median, as there are no normal values for renal T1 times yet, and although the risk assessment in patients with renal T1 times above the median is of great interest, it would also be of interest if even small changes from normal values lead to adverse outcomes.

Furthermore, research has demonstrated that advanced mapping techniques can be applied for the investigation of tissues beyond the kidney. A recently published study has demonstrated an association between elevated skeletal muscle T1 times and unfavorable outcomes in patients with heart failure with preserved ejection fraction (HFpEF) [23]. Moreover, another study demonstrated an association between liver T1 times and cardiac function parameters, as well as adverse outcomes, in an all-comer cohort of patients who underwent CMR imaging [24].

### 4.2. Risk Stratification and the Relevance of T1 Mapping in Patients with Cardiorenal or Cardiovascular–Kidney–Metabolic Syndrome

CKD is defined as an impairment of renal structure or function that persists for a period exceeding three months, with associated clinical implications [25]. The most commonly used clinical criteria for assessing renal impairment are an eGFR of less than 60 mL/min/1.73 m^2^ or a urinary albumin-to-creatinine ratio (uACR) of 30 mg/g or greater [26]. Regardless of the underlying cause, CKD is characterized by progressive and irreversible nephron loss, microvascular damage, reduced regenerative capacity, inflammation, oxidative stress, and metabolic changes, ultimately leading to renal failure and end-stage renal disease [27]. The impact of CKD on global morbidity and mortality is rapidly increasing [1,28], underscoring the urgent need for improved diagnostic and therapeutic options.

Renal fibrosis represents the typical pathological feature and end-stage manifestation of CKD. Its morphological characteristics include glomerulosclerosis, tubular atrophy, chronic interstitial inflammation and fibrogenesis, and vascular rarefaction [22]. At present, renal biopsy is the only means of determining the extent of fibrotic changes present and, thus, establishing a prognosis that goes beyond that offered by laboratory and clinical markers alone [18]. In their analysis, WU J et al. demonstrated a correlation between renal T1 times and fibrosis through the performance of renal biopsies. However, to the best of our knowledge, this is the only study that has employed renal biopsies to confirm that renal T1 times reflect fibrosis [13].

### 4.3. Limitations

It should be noted that this study is subject to a number of limitations. It is important to acknowledge that this study is a single-center post hoc analysis, which may introduce a center-specific bias. Secondly, the study did not include an evaluation of renal biopsies, which remain the gold standard for the detection of renal fibrosis or differentiation between other mechanisms, such as inflammatory diseases, which may also result in increased renal T1 times. It is possible that the presence of renal fibrosis was misclassified due to its segmental nature. Furthermore, T1 times do not permit the differentiation of the precise mechanism underlying the increased T1 times. A further limitation of the study is that 12% of the causes of death could not be identified in the medical records, which may have led to an underestimation of cardiovascular deaths and thus biased the results. Furthermore, the database lacked documentation of renal endpoints. It should be noted that, despite the exclusion of patients with cardiac amyloidosis, amyloidosis can also affect only the kidneys, which can also result in prolonged T1 times. The most commonly employed clinical criteria for the diagnosis of renal impairment are decreased estimated glomerular filtration rate (eGFR) and increased urine albumin-to-creatinine ratio (uACR). The present study aimed to examine the association between renal T1 times and eGFR, while not considering uACR, which is essential in the comprehensive assessment of CKD. Nevertheless, our study was the first to demonstrate an association between increased renal T1 times and adverse outcomes. Further research is required to substantiate these findings.

## 5. Conclusions

The present results lend support to the hypothesis that renal cortical T1 times and eGFR are closely related. Furthermore, our study results may provide additional evidence for the routine use of renal T1 mapping. In our cohort, renal T1 times were found to predict outcomes, which may allow renal T1 times to serve as a diagnostic imaging marker with prognostic implications in the future. Our findings contribute to the evidence base for the widespread use of mapping sequences in a wider range of organs and support their prognostic power. However, we would like to emphasize that our results are preliminary and need to be validated in external cohorts performing renal biopsies.

## 6. Clinical Perspectives

Given the established link between renal fibrosis, typically identified through renal biopsy, and unfavorable outcomes, renal T1 mapping may provide a more comprehensive and non-invasive approach for risk stratification in patients with cardiovascular and chronic kidney disease, beyond the scope of laboratory markers. Furthermore, there is a need to integrate non-invasive tissue characterization techniques, such as mapping sequences, into routine clinical practice, thus facilitating a more comprehensive diagnostic assessment in complex patient populations.

## Figures and Tables

**Table 1:** Baseline characteristics stratified by renal cortical T1 times.

Variables	All Patients (n = 506)	Renal Cortical T1 Times Below Median (T1 ≤ 1090 ms) (n = 255)	Renal Cortical T1 Times Above Median (T1 ≥ 1091 ms) (n = 251)	*p*-Value
		Mean ± SD	Mean ± SD	
Clinical parameters				
Age, years	61 ± 15	57 ± 16	67 ± 13	<0.001
Male sex, n (%)	268 (53%)	149 (58%)	119 (47%)	0.004
BMI, kg/m^2^	27 ± 4	27 ± 4	27 ± 4	0.887
Comorbidities				
Diabetes mellitus, n (%)	97 (19%)	38 (15%)	56 (22%)	0.076
Coronary artery disease, n (%)	52 (10%)	28 (11%)	22 (9%)	0.704
Atrial fibrillation, n (%)	153 (13%)	63 (26%)	89 (36%)	0.015
Arterial hypertension, n (%)	239 (47%)	106 (43%)	131 (52%)	0.035
Hyperlipidemia, n (%)	91 (18%)	51 (21%)	37 (15%)	0.888
CKD G1, n (%)	108 (21%)	91 (35%)	18 (7%)	<0.001
CKD G2, n (%)	183 (36%)	130 (50%)	53 (21%)	<0.001
CKD G3a, n (%)	112 (22%)	22 (9%)	92 (37%)	<0.001
CKD G3b, n (%)	65 (13%)	3 (1%)	62 (25%)	<0.001
CKD G4, n (%)	14 (3%)	0 (0%)	14 (5%)	<0.001
CKD G5, n (%)	0 (0%)	0 (0%)	0 (0%)	
Laboratory markers				
NT-proBNP, pg/mL	937 (306–2291)	540 (160–1276)	1435 (657–3327)	<0.001
Creatinine, mg/dL	1.1 ± 0.4	0.9 ± 0.2	1.3 ± 0.5	<0.001
eGFR mL/min/1.73 m^2^	70 ± 26	85 ± 22	54 ± 21	<0.001
CRP, mg/dL	0.9 ± 2	0.8 ± 1	1.2 ± 1.7	0.015
Hb, mg/dL	13 ± 2	13 ± 2	12 ± 2	<0.001
Troponin T	146 ± 545	107 ± 419	185 ± 645	<0.001
Cardiac magnetic resonance imaging parameters				
LVEF, %	57 ± 14	58 ± 14	55 ± 15	0.06
LVEF, ≥50%	338 (67%)	179 (70%)	159 (63%)	0.190
LVEF, ≥40 and <49%	57 (11%)	25 (10%)	32 (23%)	0.514
LVEF, ≤39%	62 (12%)	23 (9%)	39 (16%)	0.085
RVEF, %	52 ± 11	53 ± 10	51 ± 12	0.029
IVS, mm	12 ± 3	12 ± 3	12 ± 3	0.379
LVEDVi, mL/m^2^	84 ± 30	86 ± 28	82 ± 32	0.01
RVEDVi, mL/m^2^	81 ± 28	81 ± 22	81 ± 32	0.222
Myocardial T1 times, ms	1016 ± 46	1008 ± 43	1024 ± 47	<0.001
ECV, %	27 ± 4	27 ± 4	28 ± 4	0.001

Abbreviations: CKD, chronic kidney disease; BMI, body mass index; NT-proBNP, N-terminal pro-brain natriuretic peptide; eGFR, estimated glomerular filtration rate; CRP, C-reactive protein; Hb, hemoglobin; LVEF, left ventricular ejection fraction; RVEF, right ventricular ejection fraction; IVS, intraventricular septum; LVEDVi, left ventricular end-diastolic volume index; RVEDVi, right ventricular end-diastolic volume index; ECV, extracellular volume fraction.

**Table 2:** Uni- and multivariable linear regression analyses testing the association between variables related to log10 renal cortical T1 times.

	Univariable Analysis				Age- and Sex-Adjusted			
	β	95% CI		*p*-Value	Adj. β	95% CI		*p*-Value
Variables		Lower	Upper			Lower	Upper	
Age	0.268	0.00	0.001	<0.001				
Male sex	−0.123	−0.000	−0.001	0.006				
BMI	−0.044	−0.001	0.000	0.351	−0.030	−0.001	0.001	0.667
LVEF	−0.101	−0.000	−0.000	0.031	0.086	0.001	−0.001	0.238
RVEF	−0.094	−0.000	0.000	0.05	0.057	0.001	0.001	0.433
ECV	0.158	0.000	0.002	0.026	0.079	0.000	0.002	0.273
Myocardial T1 times	0.176	0.147	0.421	<0.001	−0.013	0.000	0.000	0.848
eGFR	−0.612	−0.001	−0.001	<0.001	−0.534	−0.001	0.000	<0.001
Creatinine	0.581	0.035	0.044	<0.001	0.532	0.027	0.042	<0.001
NT-proBNP	0.249	0.000	0.000	<0.001	0.061	0.000	0.000	0.406
CRP	0.119	−0.000	−0.005	0.090	0.075	−0.001	0.004	0.268
Hemoglobin	−0.332	−0.006	−0.004	<0.001	−0.194	−0.005	−0.001	0.009
Troponin T	−0.013	0.000	0.000	0.851	0.035	0.000	0.000	0.822

Uni- and multivariable regression analyses of parameters possibly associated with renal T1 times with unstandardized coefficients (β) and 95% confidence interval (CI). Abbreviations: BMI, body mass index; NT-proBNP, N-terminal pro-brain natriuretic peptide; eGFR, estimated glomerular filtration rate; CRP, C-reactive protein; Hb, hemoglobin; LVEF, left ventricular ejection fraction; RVEF, right ventricular ejection fraction; ECV, extracellular volume fraction.

**Table 3:** Univariable and multivariable Cox regression analyses for the prediction of all-cause death.

	Univariable Analysis					Multivariable Analysis			
	HR	95% CI		*p*-Value		HR	95% CI		*p*-Value
Variable		Lower	Upper		Variable		Lower	Upper	
Renal T1 times *	1.73	1.42	2.11	<0.001	Renal T1 times *	1.49	1.10	2.02	0.01
					Age	1.04	1.02	1.06	<0.001
					Sex	1.98	1.23	3.19	0.005
					NT-proBNP	1.00	1.00	1.00	<0.001
					Hemoglobin	0.86	0.75	0.98	0.024
					Atrial fibrillation	1.03	0.61	1.72	0.917
					Hypertension	0.77	0.47	1.27	0.304

Univariable and multivariable regression analyses of parameters possibly associated with the endpoint with hazard ratios and 95% confidence intervals (CI). Abbreviations: NT-proBNP, N-terminal pro-brain natriuretic peptide; HR, hazard rate; CI, confidence interval. * Values given for every 100-millisecond increase in T1 times.

**Table 4:** Univariable and multivariable Cox analyses for the prediction of cardiovascular death.

	Univariable Analysis					Multivariable Analysis			
	HR	95% CI		*p*-Value		HR	95% CI		*p*-Value
Variable		Lower	Upper		Variable		Lower	Upper	
Renal T1 times *	1.41	1.05	1.90	0.021	Renal T1 times *	1.42	1.05	1.90	0.021
					Sex	1.01	0.66	1.57	0.95
					Hypertension	0.95	0.62	1.47	0.823

Univariable and multivariable regression analyses of parameters possibly associated with the endpoint with hazard ratios and 95% confidence intervals (CI). * Values given for every 100-millisecond increase in T1 times.

## Data Availability

The data that support the findings of this study are available from the corresponding author on request.

## Abbreviations

ACR	Albumin-to-creatinine ratio
BMI	Body mass index
CMR	Cardiac magnetic resonance
CRS	Cardiorenal syndrome
CVD	Cardiovascular disease
CKD	Chronic kidney disease
CRP	C-reactive protein
ECV	Extracellular volume fraction
eGFR	Estimated glomerular filtration rate
LVEF	Left ventricular ejection fraction
NT-proBNP	N-terminal pro-brain natriuretic peptide
ROI	Region of interest
RVEF	Right ventricular ejection fraction
uACR	Urine albumin-creatinine ratio
